# Enhancing Reciprocity, Equity and Quality of Ethics Review for Multisite Research During Public Health Crises: The Experience of the COVID-19 Clinical Research Coalition Ethics Working Group

**DOI:** 10.1017/jme.2023.75

**Published:** 2023

**Authors:** Vasiliki Rahimzadeh, Jennyfer Ambe, Jantina de Vries

**Affiliations:** 1: BAYLOR COLLEGE OF MEDICINE, HOUSTON, TX, USA; 2: SAFE MOTHER AND CHILDHOOD RESEARCH INITIATIVE (SAMOCRI), NIGERIA; 3: DEPARTMENT OF MEDICINE, UNIVERSITY OF CAPE TOWN AND THE NEUROSCIENCE INSTITUTE, UNIVERSITY OF CAPE TOWN, SOUTH AFRICA

**Keywords:** Public Health, COVID-19, Clinical Research, Ethics

## Abstract

In this paper we report findings from a commissioned report to the COVID-19 Clinical Research Coalition on approaches to streamline multinational REC review/approval during public health emergencies. As currently envisioned in the literature, a system of REC mutual recognition is theoretically possible based on shared procedural REC standards, but raises numerous concerns about perceived inequities and mistrust.

## I. Introduction

Amidst the death and destruction of the ongoing COVID-19 pandemic, the transformative power of timely data sharing and scientific collaboration remains evident. During public health emergencies, researchers face extreme time and resource pressures to identify, contain and prevent transmissible diseases. Quality and effective research ethics committee (REC) review and approval of public health protocols involving humans likewise presents acute challenges during a pandemic. RECs — also known as institutional review boards or research ethics boards — must balance known benefits and risks to participation despite high scientific uncertainty. They also ensure research meets standards of scientific rigor, maximizes social value, and complies with applicable local, regional and international guidelines and laws.

Researchers who recruit participants and share data across multiple institutions and international borders must often obtain REC approval at every collaborating site, with few exceptions.^1^ However this site-by-site approach to REC review and approval can exacerbate procedural inefficiencies during public health emergencies, without evidence that it measurably improves participant protections.^2^ Previous public health emergencies^3^ and natural disasters^4^ often compelled changes to streamline ethics review procedures,^5^ including mandatory site-specific review^6^ and prompted development of special standards in advance of such emergencies.^7^

History and experience provided key lessons for RECs in the early months of the COVID-19 pandemic in this regard.^8^ Actions to accelerate research, streamline information-sharing, and improve governance capacities, for example, were widely supported in response to the Ebola and H1N1 outbreaks. Such streamlinining also drew criticism for the lack of uniformity and coordination among RECs operating in affected areas.^9^ A series of consultative workshops after these outbreaks revealed that stakeholders recommended RECs should develop special standard operating procedures to improve coordination among local, regional, and national bodies to respond efficiently to evolving public health evidence and guide pandemic response.^10^

In this paper, we considered the possible opportunities and challenges of a hypothetical system of ethics review mutual recognition to accelerate multinational REC review/approval during public health emergencies. We report findings from a critical appraisal of the literature on single REC review models, and summarize discussions with members of the Ethics Working Group of the COVID-19 Clinical Research Coalition (herein the “Coalition”). The Coalition was founded in April 2020 by the Drugs for Neglected Diseases initiative, the Infectious Diseases Data Observatory, and the Mahidol Oxford Tropical Medicine Research Unit. It’s mission is to “advocate and collaborate for the advancement of COVID-19 research that is driven by the needs of people in low-resource settings, and to strive for equitable access to solutions in the global response to the pandemic.” Since its founding, the Coalition has grown to more than 900 institutions and individuals from 98 countries.

Schopper and colleagues lamented that the tangible benefits of joint pre-review of Ebola clinical trials, “or at least of proactive communication among [ethics committees] reviewing the same Ebola trial protocol(s), may have been a missed opportunity to streamline the different reviews into a comprehensive review (potentially, an advantage for the researchers) and to foster dialogue and mutual learning among the different ECs/IRBs (potentially, an advantage for the ECs/IRBs).”^11^ The authors further note that blinding reviews from different committees involved in reviewing the same study “prevented exchange of views, shared approaches, to new dilemmas, and agreement on common review policies.”^12^

The ongoing COVID-19 pandemic has renewed a growing interest among researchers to address procedural bottlenecks by considering alternative systems of REC review for collaborative, multisite ethics review. Originally proposed to facilitate multisite research in genetics/genomics,^13^ ethics review mutual recognition refers to a system wherein the decisions of a competent REC are recognized by another REC based on adherence to shared procedural standards. As currently envisioned in the literature, ethics review mutual recognition purports to save both time and resources. However such efficiency gains have yet to fully account for concerns about perceived inquity and mistrust among RECs in low- and middle-income countries, nor in the specific context of public health emergencies.

In this paper, we considered the possible opportunities and challenges of a hypothetical system of ethics review mutual recognition to accelerate multinational REC review/approval during public health emergencies. We report findings from a critical appraisal of the literature on single REC review models, and summarize discussions with members of the Ethics Working Group of the COVID-19 Clinical Research Coalition (herein the “Coalition”). The Coalition was founded in April 2020 by the Drugs for Neglected Diseases initiative, the Infectious Diseases Data Observatory, and the Mahidol Oxford Tropical Medicine Research Unit. It’s mission is to “advocate and collaborate for the advancement of COVID-19 research that is driven by the needs of people in low-resource settings, and to strive for equitable access to solutions in the global response to the pandemic.”^14^ Since its founding, the Coalition has grown to more than 900 institutions and individuals from 98 countries. During publication, the COVID-19 Clinical Research Coalition was renamed the Coalition for Equitable Research in Low Resource Settings (CERCLE).

Both the literature review as well as consultations were conducted as part of a commissioned report to guide Coalition policy for REC review during public health emergencies moving forward. In the following sections, we first provide an overview of the opportunities of ethics review mutual recognition before considering the challenges (Part III) and making practical recommendations in Part IV. Our findings suggest, without being conclusive, that ethics review mutual recognition seems premature at present given the wide variation in how RECs are governed, structured, and coordinated. Discussions with Coalition members from low- and middle income countries (LMIC) furthermore highlighted that a proposed supranational system for accelerated REC/review approval accentuates mistrust — particularly when research sponsors reside in high income countries and research participants in LMICs — and substantiating the fact that enhancing trust was a necessary precondition to any such system.

We summarize lessons learnt from the ongoing COVID-19 pandemic to synthesize possible opportunities, persistent tensions, and implementation factors that may support streamlined REC review/approval procedures during the next public health emergency.

## II. Ethics Review Mutual Recognition: A Conceptual Primer

Ethics review mutual recognition refers to a cooperative system of research ethics review and approval. In a system of ethics review based on mutual recognition, a competent REC recognizes the review decisions made by another REC on a protocol application involving more than one institution. The reviewing REC, herein referred to as the “REC of record,” conducts a one-time, full board review of the multisite study application on behalf of all institutions named in the protocol with input from RECs at local sites where the research will take place and based on shared review standards. The local REC then reviews the decision; it may choose to accept the terms of the approval and serve as a participating site or can reject it, precluding participant recruitment.

Ethics review mutual recognition borrows from the legal theory of equivalence, where two regimes are equivalent when the outcomes of a shared regulatory process are the same.^15^ Equivalence has been applied as an international legal instrument to facilitate financial securities regulation, and to enable cross-border transfers of personal data, to name two contemporary applications.^16^ The goals of ethics review mutual recognition are to enhance the quality, timeliness and effectiveness of REC review and support consistency in review decisions for studies that recruit across multiple institutions and/or international borders without supplanting local review of community norms and values relevant to the study under review.

We suggest that two preconditions are needed to accomplish the goals of ethics review mutual recognition, albeit are difficult to meet simultaneously:

- procedural equivalence of REC review standards; and
- reciprocity between and among participating RECs.

Later sections of the paper will elaborate on challenges to satisfying these conditions and provide a case study example describing how they were met in part in one instance. Qualified, competent RECs can avoid inefficiencies and unnecessary duplication by standardizing procedures common to all. These procedures could include, for example, assessments of study design and scientific validity, compliance with data protection, confidentiality and security, verifying appropriate subject selection, evaluating realistic risks and potential benefits of participation, reviewing informed consent and relevant documents for comprehensiveness and comprehension. The WHO *Standards and Operational Guidance for Ethics Review of Health-Related Research with Human Participants* provides one common set of standards that counld satisfy the procedural equivalence for condition 1.^17^

While true the WHO *Standards and Operational Procedures* lacks legally binding force, they offer the nearest proxy for global consensus regarding how to procedurally apply bioethics principles to the responsible conduct of research involving humans. RECs that adopt the WHO *Standards and Operational Procedures* could theoretically have greater confidence in the decisions of other RECs insofar as they also adhere to these same standards. Moreover, the Global Alliance for Genomics and Health *Ethics Review Recognition Policy* ^18^ proposes both essential and common elements of REC review specific to research involving human health data. The purpose of the *Policy* is to “provide a common effective baseline of the ethics review process for multi-jurisdictional research”^19^ that, when adopted, could inspire greater confidence in the compatibility of two REC’s decisions.

Systems based on mutual recognition then leverage shared procedural standards to establish reciprocal agreements between and among RECs. Establishing reciprocity following a determination of procedural equivalence together comprises the two-pronged approach that a system of ethics review mutual recognition applies to reduce administrative burden and delays for otherwise ethically compliant research. It is important to emphasize that the locus of reciprocity in ethics review mutual recognition is procedure, not substance. Harmonizing the latter would require that all RECs accept, interpret and apply research ethics principles in the same ways. Requiring this level of uniformity is highly problematic as it disrespects the plurality of moral traditions that exist across cultures and societies and risks being hegemonic.

Importantly, reciprocal agreements pursuant to condition 2 do not supplant authorization from local sites, nor do they sidestep community representation that local RECs bring to their own review processes (see *Section* *III* for a case example). That is, an RECs may decide at the point of providing local authorization that the reviewing REC’s approval is based on an ethical analysis that is too permissive or too strict to align with the values and priorities of the local community. Instead, systems based on ethics review mutual recognition place a higher premium on deliberation of substantive ethical issues that a study presents and which local RECs are best positioned to address (e.g. coherence with social, cultural and ethical values of prospective research participants, research feasibility, recruitment approach).

## III. Challenges and Opportunities for Ethics Review Mutual Recognition in LMICs

The first author (VR) led informal discussions with twelve members from Coalition and their colleagues from November 2020-January 2021 with the goal of informing Coalition guidance on multisite ethics review in pandemic settings. The persons she spoke with were also members of various RECs from North and South America, the Caribbean, Europe, Eastern Europe/Central Asia, and Africa. Discussions were framed around broad topics and member experiences related to the quality and timeliness of multisite ethics review during the COVID-19 pandemic in their home countries, including:

- Existing organizational infrastructures for single- and multisite REC review, applicable laws/regulations and REC operations:
- Processes and procedures for reviewing multisite research proposals and specific REC standard operating procedures for public health emergencies:
- Modifications, if any, to REC procedures during the COVID-19 pandemic: and
- Perceived challenges and opportunities of pursuing a global system of ethics review mutual recognition and its impact on perceived quality and effectiveness of REC review.

The Stanford University Institutional Review Board deemed the activities to be exempt from review.

Findings from these discussions suggest that ethics review mutual recognition affects at least three important stakeholder relationships between the reviewing REC of record and local REC committees, researchers, and participants. Key mediating factors influencing whether, and how to pursue a multilateral system of mutual recognition in ethics review differed extensively by country and region. Common themes emerged in discussions about practical challenges, including a) lack of conceptual clarity on the protected roles of local RECs, b) the heterogeneity of regulatory environments within which RECs currently operate, and c) perceived inequities in North-South research collaborations. We elaborate on these three aspects below and provide examples where applicable.

### Protecting Local REC Review and Decision-Making Authority

The importance of local context to REC review is well characterized in the literature.^20^ Scholarship on the most effective methods for incorporating local knowledge into REC review, however, varies widely.^21^ Attention to ethical, legal, and social issues relevant to proposed research studies have been traditionally served by localizing the review process amidst growing criticism about whether such localization measurably improves participant protections. Arguments against centralizing REC review generally falls into two categories. Ceding full board review to a centralized or single REC either wholly or in part can (1) compromise protections for local participants because the REC board of record lacks insight into local values, interests and priorities; and 2) robs local RECs of institutional autonomy they would otherwise maintain in single-site studies.

Sociologist Adam Hedgecoe was among the first to empirically study the tradeoffs of local versus centralized REC decision-making, claiming “local knowledge plays a vital yet largely overlooked role in how RECs make decisions.”^22^ Chair of the Médecins Sans Frontières Ethics Review Board, Raffaela Ravinetto, and colleagues confirm the protective benefits of maintaining a system of “double ethical review,” when RECs in both the sponsoring and host country conduct independent reviews of a multisite study.^23^ Advocates for double ethical review contend it “de facto strengthens the protection of the study participants and their communities…Despite their universal character, ethical principles governing clinical research need to be translated into rules, procedures and practices, which may significantly vary among countries and regions.”^24^

Studies involving REC members in Europe and North America similarly endorse local review of a multisite study when it includes international partners and where sociocultural differences warrant local input.^25^ According to another study of REC members in The Netherlands, RECs manage bureaucratic tensions in the review process based on resource availability and institutional context. These differences help RECs develop what the authors call “situational authority” over when, how, and which studies proceed^26^ and was a common theme identified in discussions with REC members represented in the COVID-19 Coalition when deciding the merits of centralizing multisite review of vaccine-related trials in the early stages of the pandemic.

Implicit in many of the above arguments rejecting single REC review are assumptions about how local RECs obtain, retain, and effectively apply local knowledge to ensure participant interests and values are duly served in the research. For instance, it presumes that the professional elites that dominate ethics committee deliberations adequately understand and represent the concerns of lay members of the population that they serve.

Proponents of single REC review challenge the assumption that full board review at each participating site is the most effective way for local values to be represented in the review process. For instance, Klitzman and colleagues^27^ argue that conditions in the local context could be adequately captured in reviews conducted by a single REC of record. They identify four locally specific insights that any centralized body would need to have in order to conduct a quality and effective review:

- Cultural and linguistic characteristics of prospective participants;
- Geographic and socioeconomic issues;
- Knowledge about specific researchers; and
- Institutional differences across participating study sites.

Furthermore, Emanuel and colleagues claim that there is insufficient evidence to “substantiate the value of […] local knowledge or whether it can only be — or is best — gained through institution-based review.”^28^ Put another way, one critical question is whether current models for incorporating local knowledge into reviews — namely by requiring site-specific review — successfully achieves the goal of community representation, and whether this purported benefit is at the expense of time-sensitive research during public health emergencies. Workflow improvements, such as standardized application and review forms,^29^ online submission and review portals, as well as software have been identified as recurring needs to streamline RECs process applications,^30^ monitor progress and more directly incorporate input from community members on the REC at each stage in the review process from submission to continuing review.

Other scholars advocate for a new conceptual framework for multisite ethics review. For example, Townend and Dove propose RECs take a “sounding board” approach when serving as the board of record for international, multisite studies.^31^ The sounding board model places the onus on researchers to ensure local interests have been adequately considered and plans for protecting local participants are socioculturally appropriate. This model “obviates the need for a harmonised substantive ethics, which we believe is an intractable challenge” and instead emphasizes harmonization of those procedural elements common to all RECs.

Yet key to successful adoption and implementation of mutual recognition is, as we argue, empowering RECs to be responsive to local values while optimizing procedural efficiency in the review process. Discussants consulted for this report emphasized the point that local RECs should be directly involved in reviewing all multisite studies and expressed grave concern that a proposed system of ethics review mutual recognition would eliminate local input from decision-making. When asked how RECs modified pre-pandemic procedures to meet the new demand for multisite studies during the pandemic, each discussant acknowledged that the volume of new COVID-related protocols stressed local REC resources and reported that centralizing some component of the review with local authorization occurred. Discussants were also unclear how local input would be integrated into the final authorization of a multisite or multinational study, what authority local RECs would maintain if any, and the extent to which ethics review mutual recognition would affect community representation, more broadly. These are important concerns that will need to be addressed in the development or design of any system that proposes to centralise some of the REC review functions.

### REC Organization

Ethical principles guiding responsible conduct of research involving humans emerged primarily in response to a troubled history of exploitation and abuse. While some of the earliest RECs operated in hospitals and research institutions in the 1960s, it was not until the early 1970s that the proposal for institutionalized RECs was written into the second revision of the Declaration of Helsinki and made a regulatory requirement for human subjects research in the UK, US, and Canada. The proposed constitution, structure, and oversight operations of institutional RECs remain largely unchanged. While membership may be broad, core REC members have professional expertise in fields such as ethics, medicine, law, and science, and many committees consult external experts to guide their review of studies with novel study designs, for example, or perhaps to gather informed input on how the study procedures align with the values of a prospective participation population. Importantly, community representatives help to represent the values and interests of the local population and their membership on the REC is mandatory in many countries.

**Figure 1:**
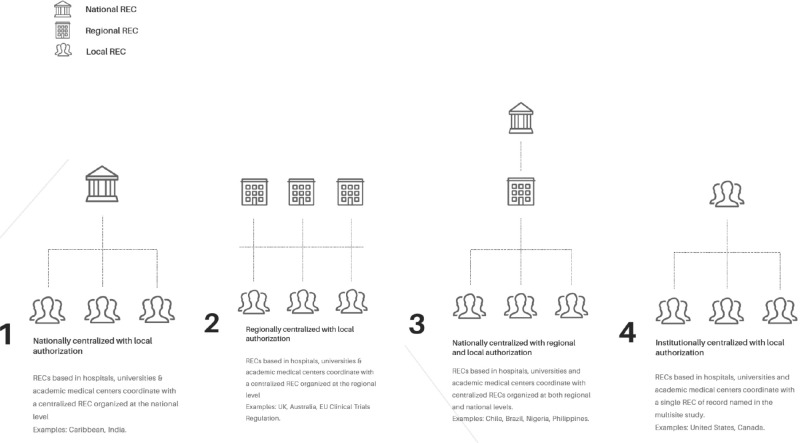
Four models of single or centralized ethics review for multisite studies adopted by RECs during the pandemic and represented in the Ethics Working Group of the Global COVID-19 Research Coalition.

Social, economic and political stability are, as some authors argue,^32^ key enabling factors for sustaining a robust REC system that greatly impacts the regulatory and institutional environments within which RECs operate. Figure 1 demonstrates various modalities for how RECs are embedded within these environments. RECs can be governed by national or regional authorities or by international organizations such as Médecins Sans Frontières and World Health Organization Headquarters and operate under rules outlined in ministerial mandates (e.g. health, research, education or equivalent). Some countries have a single national REC that serves as the official reviewing authority for all research involving humans performed in a country;^33^ others have many RECs. In some countries, the national REC only reviews certain types of research. Some RECs operate independently from institutions, but apply rules set forth by a country’s national health system. Finally, regional RECs may comply with national guidelines for human research protections but receive funding from or be mandated by a regional authority. Only in recent years have some national agencies introduced centralized or single ethics review for studies in which recruitment spans multiple institutions, including rapid response committees deployed during public health emergencies.^34^

Centralized committees may rely on institutional RECs to assess locally contingent elements of a protocol. These assessments can be both pragmatic and normative. Local REC review may be best positioned to evaluate the availability of human and material resources necessary to carry out study interventions and determine whether participant recruitment is appropriate and feasible. The arrangements by which scientific collaborations have traditionally been set up between researchers in the Global North and South can further distance researchers from the individuals and communities involved in the research. This distance is literal in that powerful information technologies allow researchers physically located in different geographies to collect, analyze, and share research data more than ever before. This distance is also metaphoric in that researchers may be culturally ignorant of the values and priorities of communities they recruit to the study, potentially affecting how effectively the REC of record can protect the interests of local communities. The concept of cultural distance is relevant to the success of ethics review mutual recognition insofar as members of the RECs of record need to demonstrate knowledge of, and sensitivity to the norms, values and priorities of communities within which local RECs are embedded and serve.

Many discussants articulated that the diversity in regulatory environments is a primary reason why establishing reciprocity among RECs — the second of two preconditions needed to support ethics review mutual recognition — is likely unrealistic and incompatible with applicable rules and laws in their home countries. For example, establishing reciprocity requires amendments to existing rules/laws for researchers and healthcare professionals because RECs are co-regulated with healthcare delivery in some countries, such as Brazil. In Nigeria and Ghana, discussants identified similar challenges in that adopting ethics review mutual recognition would require one system of governance for two otherwise distinct professional sectors within the Ministry of Health. All of these challenges suggest that the introduction of a system of ethics review mutual recognition is neither straightforward nor easily implemented.

Our discussions suggest that ethics review mutual recognition should be better integrated within existing legal and regulatory systems governing research involving humans regardless of their location if RECs are to feasibly implement it. However, such integration remains both a regulatory and logistical barrier to establishing reciprocity. A superior challenge arises with the introduction of a system of ethics review mutual recognition that spans the international arena. In the next section of the paper, we will speak specifically to one dimension of international collaboration that our discussants identified as a major obstacle to regulating ethics review internationally, namely trust between people from different nations.

### North-South Equity Relations and Manifestations in REC Review

The viability of ethics review mutual recognition is hindered by a trust problem among RECs working in LMICs when reviewing applications for sponsored research from well-resourced institutions in high income countries (HICs).^35^ Scholars corroborate this lack of trust and emphasize the essential role that local REC review plays in representing not only communities but also national values, interests and priorities.^36^ Enhancing opportunities for trust-building among RECs is a prerequisite to any functioning REC system based on mutual recognition.^37^ There are also often other tensions between government, researchers, and communities that inevitably affect the durability of such economies of trust.^38^ Finally, while measures of REC review quality should include normative goals such as equity and inclusion, and performance goals like effectiveness and efficiency, recent analyses suggest that they rarely do.^39^

Most published articles available on centralized ethics review are primarily authored by REC actors/scholars based in high income countries in North America, Europe, and Australia. Absent a more globally balanced representation of REC experiences during the pandemic, the literature offers a skewed understanding at best about the merits and pitfalls of ethics review mutual recognition. Many discussants expressed healthy skepticism to this effect. The anticipated gains in efficiency afforded by centralizing reviews did not outweigh concerns about equity relations between RECs of record and local RECs. Rather, they precipitated fears that ethics review mutual recognition would further limit opportunities for meaningful engagement and community prioritization in REC decision-making. This issue was particularly salient when discussing North-South research collaborations.

There was broad concern about the extent to which international research conducted in LMICs continues to entrench unequal power relations characteristic of colonialism, corroborating findings from prior research.^40^ While willing participants based in LMICs were enrolled in vaccine trials, reports indicate they have not equitably benefited.^41^ Few commitments by HICs to support global vaccine distribution has reinforced a perception that internationally, financial interests of wealthy countries take priority over values such as solidarity and health equity needed to achieve global herd immunity.^42^ Scholars from the global South have also argued that commitments as to vaccine research and equitable distribution were motivated not by genuine solidarity but rather by threats to the health and welfare of people in wealthy countries. Rate and trends of vaccine distribution and deaths are suggestive of gross inequities,^43^ which one retrospective modeling study found contributed to at least 1.3 million excess deaths worldwide.^44^ Global vaccine trackers estimate that 12.8 billion doses of COVID-19 vaccines have been administered globally as of November 2022. Still more than 80 percent of people living in LMICs have yet to receive even one dose.^45^ LMICs now lead the world in COVID-19 deaths per 100,000, and countries where fewer than 20 vaccine doses have been administered per 100 are located on the African continent.^46^ Suspicions about the motivations of researchers and institutions based in wealthier countries, and experiences of scientific marginalization, mean that many scholars based in LMICs are deeply sceptical about efforts that may seem to eradicate the limited protections that exist in scientific research. From this perspective, national or local ethics review provides at least some safeguard against exploitation. A system of multinational ethics review needs to accommodate such concerns and actively promote technologies and approaches that are trustworthy and that promote accountability.

A second challenge confronting collaborative, multinational ethics review for several discussants was which ethics should govern transcultural clinical research.^47^ Their perceptions differed from some reported in the literature whereby local RECs are seen to raise administrative (non-ethical) issues more frequently in ethical deliberations about biomedical research protocols.^48^ Indeed, conceptual and theoretical frameworks underpinning today’s REC procedures and operations appeal to principlism and other predominantly Western paradigms of ethics. In so doing, indigenous and non-Western values with respect to responsible conduct of research involving humans could be discounted without opportunities to co-construct culturally-competent practices for REC review.^49^ Barchi and colleagues highlight the effects of resulting resource- and power asymmetries between RECs in the Global North and South.^50^ The authors claim that oversight of international research is “fertile terrain for mistrust” when “U.S. IRBs assume that host-country IRBs are unable to conduct adequate reviews, and the latter are skeptical that U.S. IRBs will be sensitive to their national concerns or place the needs of a local population above the imperatives of the U.S. research enterprise.” ^51^

Against such concerns, and where researchers or institutions have little opportunity to avoid exploitative research practices, ethics review can be co-opted as a source of resistance against the colonizing tendencies of international research.^52^ We propose, as others have,^53^ that theories of reparative justice^54^ could proactively inform approaches to enhancing equity in REC review by, for example, incorporating specific REC review related to benefit sharing,^55^ mandatory compensation for research-related injuries,^56^ and potentially adding retrospective REC review of the social impacts the research had on participant communities.^57^

### REC Communication Lost in Translation

REC coordination and communication are well-evidenced problems which are exacerbated in the context of multisite, collaborative research.^58^ Many practical and logistical challenges constrain the feasibility of implementing a system of mutual recognition for multisite, public health research in pandemic settings. First, delineating responsibilities could prove challenging between reviewing RECs of record and local authorizing RECs. Second, the absence of a central database listing all operating RECs globally makes it difficult for researchers to seek support from local RECs when preparing their protocol application. Third, RECs based in under-resourced settings often rely on paper-based filing for application intake and processing, whereas those in wealthy countries often employ inaccessible and costly submission and ethics review portals, which can complicate document sharing across RECs and participating institutions.

Discussants with direct experience reviewing multisite/national studies related to COVID-19 in countries with and without centralized models of ethics review described significant issues in coordination and communication between reviewing RECs, local RECs, and researchers. Because communication was poor between RECs from the same region and in pre-pandemic times, discussants expected such challenges to scale when RECs from different countries as “research ethics committee would not wish to regard itself as being subservient to another, or to give up control over its current areas of jurisdiction.”^59^ Saxena and colleagues underscore the negative effects that territorial local RECs may have on communication and coordination. They argue that since RECs do not proactively communicate with other RECs evaluating the same protocol, there is a

missed opportunity for a mutual learning process among ethics committees from different contexts. Proactive communication would offer the opportunity to build collaborative partnerships among the committees, beyond partnerships between clinical researchers. Such committee partnerships could provide a space where agreement is reached on common ethical practices and standards…where more efficient models and schedules for international ethical reviews are built and the submission schedule is optimized…and where cases of conflicting opinions in different countries are resolved.^60^

Continued efforts to strengthen communication, reporting and collaborative partnerships among RECs and their governing authorities across countries (see for example the work of Fogarty International^61^) is a necessary first step to enhancing willingness among RECs to work together.

## III. Opportunities for Ethics Review Mutual Recognition in LMICs

Procedural streamlining of particular elements of REC review that are repetitive (e.g. quality control of REC applications for consent forms, description of study objectives, scientific methodology, description of benefits and risks) and demonstrate a high degree of consensus (e.g. recognizing special protections for participants from vulnerable classes) are possible based on international norms, conventions (e.g. Declaration of Helsinki) and guidelines (e.g. WHO *Standards*, CIOMs). Trust may be fostered between institutions within and between countries which share similar concerns about equity and exploitation. Reforming REC procedures to better support rigorous, and more facile scientific collaboration with researchers and community partners alike can lead to broader inclusion of populations currently underrepresented in research and, ideally, broaden the distribution of anticipated research benefits.

### AVAREF Case Example

The AVAREF (African Vaccine Regulatory Forum) joint review model is one example from which the REC community can learn how a system of ethics review mutual recognition could potentially scale for multisite clinical trial research. The AVAREF joint review model remains the only operational, extra-jurisdictional system of ethics review and approval based on mutual recognition to our knowledge, and motivates its inclusion as a case study of interest here. The AVAREF model also practically demonstrates how regulators across countries can achieve the two conditions under which we hypothesize ethics mutual recognition is most likely to succeed, namely by adopting procedural standards (condition 1) for REC review, and establishing reciprocity between participating sites that recognize the decisions of the joint committee (condition 2).

AVAREF was established by the WHO in 2006 to enhance human research protections, REC capacities, and biomedical research collaboration on the African continent. It has since issued nine consensus guidelines to guide quality and effective review of clinical trials for vaccine development, specifically. Representatives to AVAREF from 55 member states reviewed and approved the guidelines in October 2019, marking a “shift to standardized clinical trial applications and assessments, and proof of ongoing harmonization initiatives on the continent, which will ultimately lead to shorter timelines for product development.”^62^ The AVAREF guidelines outline processes and procedures for joint review of multisite/multi-national vaccine trials, including how to plan, organize, and conduct joint reviews to reduce redundancies and improve efficiency of multisite clinical trial applications in Africa. Before a joint review committee convenes, the trial sponsor agrees to procedural, transparency, and communication requirements. Notably, a candidate medical product such as a vaccine under review must be deemed to have high public health value to African countries from the perspectives of participating representatives serving on the committee. Next, representatives from participating sites convene a joint committee meeting. They apply equivalent procedural standards for REC review of vaccine trials (condition 1) through AVAREF templates, tools, criteria as well as WHO *Guidelines on Clinical Evaluation of Vaccines: Regulatory Expectations*. Subject-matter experts and a neutral proctor are invited to the meeting in addition to participant observers. The joint committee issues a final decision which is recognized by public health authorities from countries represented on the panel (condition 2). Local RECs retain the authority to accept the joint committee’s review or to opt out of the study/trial.

The AVAREF model demonstrated success most recently in July 2020, when it was leveraged to review trials investigating the safety and efficacy of COVID-19 vaccine candidates. One goal of the AVAREF COVID-19 Action Plan was to accelerate research and development of effective therapeutics and diagnostics, and it committed to finalizing its joint committee ethics review within 10 working days. The first joint review committee of a multinational COVID-19 vaccine trial on the continent included reviewers from 14 African Member States, 12 of which were able to deliver a decision in less than 25 days. AVAREF also elaborated on challenges encountered in this multinational effort, which included reasons for review delays. The organization reported that “delays in ethics decisions and in some cases by National Regulatory Authorities (NRAs) were responsible for the unmet timelines. Another major cause of delay was additional queries raised by some countries outside the joint review process, in some cases ignoring adequate responses to queries which sponsors had provided during the joint review.”^63^

The AVAREF joint committee model demonstrated practical advantages to site-specific REC review. It allowed for representatives of participating sites to collectively review safety and efficacy data for vaccine candidates, and to openly discuss relevant ethical issues participating sites were likely to face in participant recruitment, retention, and post-trial access to vaccines. The model also advantaged trial sponsors, who were able to submit a single protocol for review that could then be replicated across participating sites, enhancing rigor and protocol consistency. Representation from trial sites as part of the joint review process is a key feature of the model that helps to ensure the protocol aligns with local values and resources, and respects members’ authority to opt out of the trial if they are maligned.

## IV. Practical Recommendations to Support REC Trust and Capacity Building

Findings from our discussions with Coalition members suggest, without being conclusive, that recommendations for how to operationalize ethics review mutual recognition during public health emergencies are premature. Rather, improving trust among RECs is a pre-requisite to better functioning ethics review systems generally, and towards fostering inter- and intra-national research collaborations that are equity-enhancing.

Despite skepticism of ethics review mutual recognition as a foreseeable policy option in the near term, our discussants identified practical ways to encourage trust building among RECs globally. Such trust building activities are incremental steps towards raising the standards for cooperation and quality REC review everywhere. These practical recommendations are provided in Box 1.

Moving forward, the international REC community should consider creating professional REC networks and accessible forums^64^ that encourage sharing of knowledge and lessons learned. Furthermore, the international research community – possibly as galvanized through WHO — should make more effort to maintain an active registry of RECs operating in different countries worldwide. A registry could also be created to make decisions available for review by other RECs as a helpful way to build review capacities. Finally, more empirical research that evaluates REC review outcomes and processes (see for example^65^) is needed to inform evidence-based decisions about effective REC policy. Also needed is investment in technologies or platforms for collaborative reviews premised explicitly on promoting trust between partners. Such technologies or platforms need to be geared specifically to allow institutions to continue to rely on the REC review process as reducing institutional risk by integrating ways of archiving and recording the review process and all documents associated with it. Specifically, current suggestions for fostering multi-site ethics review seem to want to foster relations of trust between committees, ignoring the fact that it is equally important that the institutions that host the committees also agree and trust each other.

Box 1. Practical recommendations for supporting REC trust and capacity building in low- and middle-income countries before, during and after public health emergencies.

1. Consult standardized review criteria to support quality, consistent, timely and effective REC review of study protocols during public health emergencies.
2. Treat each multisite/multinational review as an opportunity to empirically evaluate internal processes and procedures and evolve best practices.
3. Making prior REC decisions for a given protocol available for review by participating RECs.
4. Maintain an international registry of operating RECs that is regularly updated and managed.
5. Clearly delineate responsibilities between reviewing RECs of record and local RECs representing participating sites.
6. REC members should be encouraged to widely disseminate and share their experiences, particularly when review procedures have been adapted during crisis.
7. Coordinate across national professional REC organizations, networks and forums to facilitate knowledge sharing.

In countries working to attract research investment through fortifying REC capacities, and in countries hoping to correct the gross underrepresentation of diverse populations in biomedical research, the pandemic has presented opportunities to take stock of existing REC infrastructures and consider improvements where feasible. Perspectives shared through consultative discussions with global REC stakeholders, suggest there is broad agreement that multisite REC reviews should be conducted by competent experts, with community representation, with local interests in mind and in a transparent, independent manner. However, discussants disagreed on the role of local RECs. There remains broad heterogeneity of regulatory environments within which RECs currently operate, and the perceived inequities in North-South research collaborations tempered enthusiasm for a system based on mutual recognition conceptualized in the literature. Our discussions with REC stakeholders thus highlight a policy tension where the desires for a procedurally robust, and nimble ethics review system are constantly renegotiated in light of deep suspicion of research(ers’) objectives. Future initiatives to operationalize ethics review mutual recognition should therefore be cooperative, bottom-up and commit health services and policy research to empirically test whether it is fit for purpose.

## V. Conclusion

The ongoing COVID-19 pandemic has placed into stark relief the importance of scientific collaboration and engaging in responsible conduct of research that results in real benefits to affected populations globally. The pandemic also served to “stress test” countries’ abilities to meet the global demand for rapid and rigorous public health research to guide pandemic responses without compromising quality and effective REC review.

The actual costs and benefits of a supranational system of ethics review mutual recognition are largely theoretical. In the meantime, we promote a reconceptualized vision of ethics review mutual recognition that first builds trust, treats local REC input as a necessary component to quality and effective multisite review, and balances this need with the exceptional time and resource constraints during public health crises.

## Note

This article is part of an Epidemic Ethics/WHO initiative which has been supported by FCDO/Wellcome Grant 214711/Z/18/Z.
